# Biomechanical Analysis of the Change of the Metatarsophalangeal Joint’s Center of Rotation After Weil and Triple Weil Osteotomies: A Comparative Cadaveric Study

**DOI:** 10.7759/cureus.21866

**Published:** 2022-02-03

**Authors:** Dimitrios Bougiouklis, Minos Tyllianakis, Despoina Deligianni, Elias Panagiotopoulos

**Affiliations:** 1 Department of Orthopaedics and Traumatology, General Hospital of Ammochostos, Paralimni, CYP; 2 Department of Orthopaedics and Traumatology, University General Hospital of Patras, Patra, GRC; 3 Department of Mechanical Engineering and Aeronautics, University of Patras, Patra, GRC; 4 Department of Orthopaedics and Traumatology, University of Patras, Patra, GRC

**Keywords:** biomechanics, metatarsophalangeal joint, interosseous muscles, triple osteotomy, weil osteotomy, metatarsalgia

## Abstract

Background

The aim of the present biomechanical study on cadavers was to determine both the center of rotation of the metatarsophalangeal joints and the position of the tendons of the interosseous muscles after the Weil and triple Weil osteotomies, and to compare these parameters in order to clarify the pathogenesis of dorsal stiffness and floating toe.

Materials and methods

Seven fresh-frozen cadaveric feet were utilized. After completing the preparation of both the plantar and the dorsal surface, we performed the dissection of the entire second, third and fourth rays, and each ray was fixed to a wooden wall mounted on a movable frame. The biomechanical analysis was based on an equilibrium system made of pulleys, threads, and variable weights. Geometrical analysis of both osteotomies and fluoroscopy was used to determine the initial and final metatarsophalangeal joint’s center of rotation, as well as the change of interosseous muscles position.

Results

On comparing the results of the findings, we noticed that after Weil osteotomy, the metatarsophalangeal joint’s center of rotation was proximally and plantarly displaced by 3.5 mm compared to the control group, and by 3.7 mm in comparison to the triple Weil osteotomy group. In the latter, the center of rotation was displaced by 0.817 mm compared to the control group. Furthermore, after the Weil osteotomy, the position of the interossei tendon was above the metatarsal longitudinal axis.

Conclusion

In cases where a metatarsal shortening of 5 mm or greater is desired, the Weil osteotomy causes a statistically significant plantar displacement of the metatarsophalangeal joint’s center of rotation, compared to cases where triple Weil osteotomy is performed.

## Introduction

The term metatarsalgia is often used to refer to the generalized or localized pain of the forefoot in the metatarsal head’s area [1]. In fact, it is a descriptive rather than a diagnostic term that includes a number of clinical conditions of different etiology. Indeed, the term metatarsalgia is referred to an acute or chronic pain that affects one or more metatarsophalangeal joints and is caused by damage of anatomical structures (bone, articular cartilage, capsule, ligaments, vessels, and nerves) [2]. Surgical treatment of metatarsalgia is indicated when the conservative measures fail to alleviate symptoms.

The Weil osteotomy was described by LS Weil in Chicago (1985) and familiarized in Europe by the French orthopaedic surgeon S. Barouk. Being a simple surgical technique with high rates of union and predicted results, it quickly became the most commonly used procedure in the treatment of third-rocker or propulsive metatarsalgia [3, 4]. However, this procedure is not without the possibility of complications, with the floating toe being the most important one [5]. In 1998, Maceira et al. described the triple Weil osteotomy, based on the observation that the plantar displacement of the metatarsal head, as a result of the Weil osteotomy, is the primary cause of the floating toe [6]. With this modified technique, the Spanish orthopedic surgeon attempted to achieve a more controlled and coaxial to the shaft shortening of the metatarsal; the elevated head can discharge the affected ray in patients with third-rocker metatarsalgia and in some selected cases of second-rocker metatarsalgia [7-9].

The aim of the present comparative cadaveric study was to determine the change of the center of rotation of the metatarsophalangeal (MTP) joints through geometric analysis of the Weil osteotomy and of its modification proposed by Maceira et al. Taking into account the etiology of the floating toe, the change of the metatarsophalangeal joint’s center of rotation will have as a result the change of the position of the tendons of the interosseous muscles, which will lose their normal actions, and they will become extensors rather than flexors of the MTP joint.

## Materials and methods

Specimens

**Figure 1:**
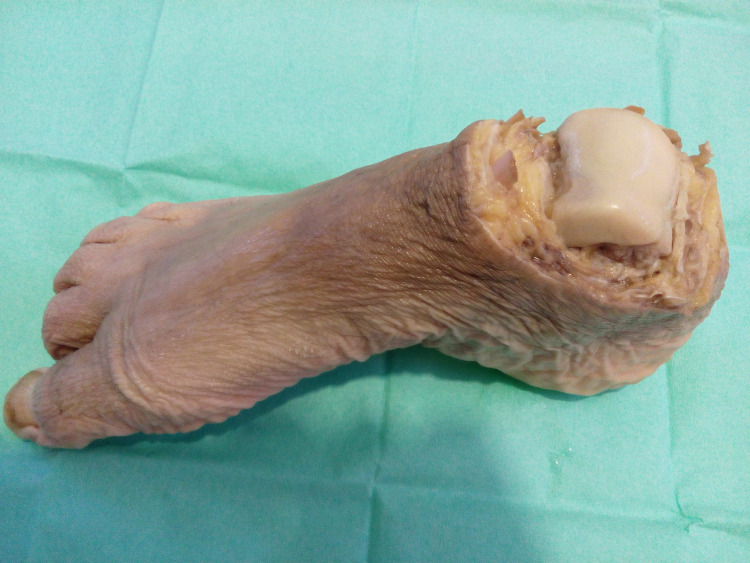
The human foot specimens used in the study were disarticulated at the level of the ankle joint. Tendons biomechanical properties were maintained for three weeks, storing the feet at night at -4 degrees Celsius.

The study was conducted at the University of Patras and was institutionally approved by the Medical School under Protocol No. 2004/12848. A total of seven fresh-frozen cadaveric feet were utilized in this study. All feet had been amputated from patients who were hospitalized at the University General Hospital of Patras and at the General Hospital of Ilia, Nursing Units of Pyrgos, because of a circulatory disorder. All specimens were dissected at the level of the ankle joint, taking care to maintain intact both the tendons of extensor digitorum longus and the tendons of flexor digitorum longus at the level of the talus (Figure 1).

All feet were immersed in a mixture of Mercuryl (a trade name for a colorless water-soluble bactericidal based on phenylhydrargyrum boricum and isopropanolum) and glycerol, which enabled them to be stored for three weeks, storing them at night at -4^o^C. During this time special care was taken to keep them constantly fresh.

One foot remained for a period of eight months in 4% formalin solution and therefore, due to the shrinkage and hardening of the joint capsules and ligaments, it was used only for preliminary experience in anatomical dissection.

Anatomic preparation

Anatomical preparation of both the dorsal and the plantar surface was performed on all feet. On the dorsum of the foot, after lifting the extensor digitorum longus tendons as well as the peroneus tertius tendon, which lie immediately subcutaneously, free access to the extensor digitorum brevis muscle was gained. It is worth noting that the extensor digitorum brevis sometimes runs independently, without joining the extensor digitorum longus, to form, by itself, the lateral slip of the extensor apparatus of the lesser toes. The extensor digitorum brevis muscle was detached at its origin along the surface of the calcaneus and from the antero-lateral side of the inferior retinaculum and readily raised off the underlying structures, exposing the capsule of the metatarsophalangeal joints, the metatarsals, and the interosseous muscles lying in-between the latter. The tendons from both dorsal and plantar interosseous muscles course distally and dorsally to the deep transverse metatarsal ligament, in contrast with the lumbricals muscles which do so plantarly, to reach the metatarsophalangeal joint and been inserted in the plantar aspect of the proximal phalanx and plantar plate [10].

**Figure 2:**
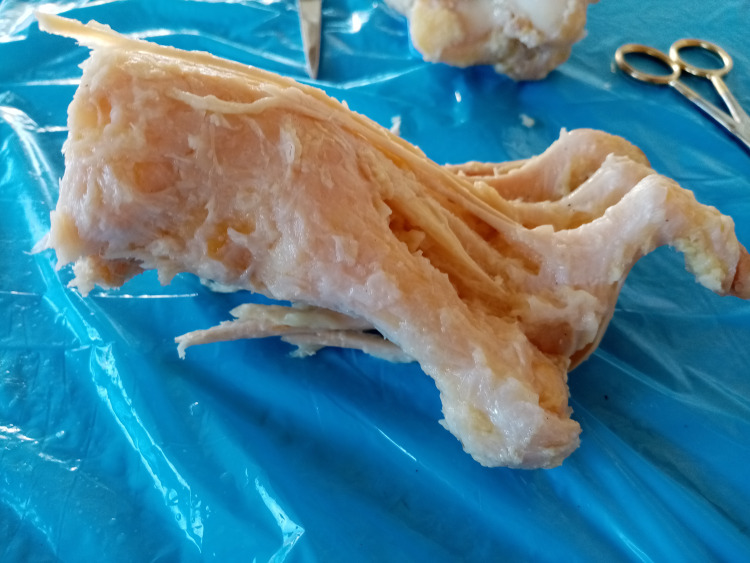
The tendons of the dorsal and plantar interosseous muscles insert onto the base of the proximal phalanx and flex the metatarsophalangeal joints. Some fibers of these tendons also insert onto the plantar plate.

On the plantar area of the foot, we proceeded to the anatomical preparation of all the three layers until the identification and determination of the course of the tendons of the three plantar interosseous muscles, which arise on the medial aspect of the delimiting metatarsal bones in its inferior surface. Given their location, plantar to the center of rotation of the MTP joint, the tendons of the interosseous muscles flex the joint by countering the extensor function and stabilizing the extensor apparatus [11, 12] (Figure 2).

After completing the preparation of both the plantar and the dorsal surface, we performed the dissection of the entire second, third and fourth rays at the level of the tarsometatarsal joints. The term "ray" on the foot, anatomically means the complex of metatarsal bone with the toe. On a human foot, there are five rays. In our study, we included six feet. From each of them, we amputated en bloc the second, the third, and the fourth ray; so from six feet, we obtained: six 2nd rays, six 3rd rays, and six 4th rays: a total of 18 rays to study. In our study, we had two groups, each of them consisting of three feet, or nine rays (three 2nd rays, three 3rd rays, and three 4th rays). Therefore, the en bloc excision of each ray included the metatarsal shaft, the metatarsophalangeal joint with the plantar fascia, the toe, and all the tendons (extensors, flexors, interosseous, and lumbricals). Thus, for the comparative study, nine rays for each osteotomy (three 2nd, three 3rd, and three 4th rays) were obtained.

At this point, the cross-sectional area of tendons was obtained by clamping the tendon between two glass plates (microscope slides) of known thickness and the jaws of a digital Vernier caliper. Simultaneously, the width of the squeezed tendon (the length of which did not increase) was measured with a scale through the transparent glass to the nearest ½ mm.

Biomechanical testing

**Figure 3:**
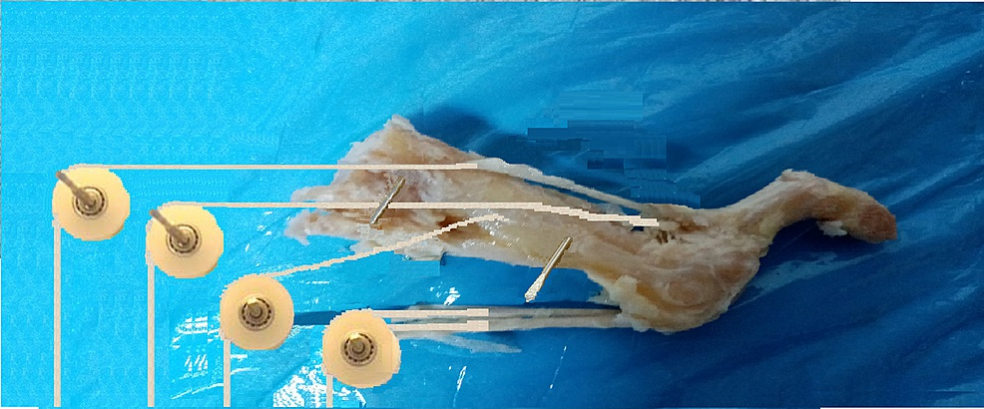
Τhe fixation on the wooden wall of each ray was done with two Steinman pins and representing the normal anatomical inclination of the metatarsal relative to the ground. Threads were tied at the ends of the tendons. Variable weights were applied to the free end of the threads. The pulley system served to balance the metatarsophalangeal joints.

After that, each ray was fixed, via Steinmann pins, to a wooden wall mounted on a movable frame. At each ray, we used two points of fixation on the wooden wall. The first one was at the proximal edge of the metatarsal and dorsally in relation to the diaphyseal axis. The second one, in a distance of 10 mm distally of the center of the metatarsal diaphysis and plantarly in relation with its longitudinal axis. The fixation of the second ray to the wall was done with an inclination of 15^o^ to the ground level, while the fixation of the third and fourth ray was done with an inclination of 10^o^ and 8^o^, respectively. An attempt was made, in this way, to simulate the normal anatomical position [13]. Threads were tied at the ends of the extensors, flexors, interosseous, and lumbrical tendons. The threads were passed through pulleys, mounted on the wall with pins in order to simulate the force which, under normal conditions, is applied by the various tendons in the metatarsophalangeal joint (Figure 3). It is noted that, in relation to the horizontal plane, the tendons of the interosseous form an angle equal to 35 degrees [14]. Variable weights were applied to the free end of each thread until equilibrium was achieved in the system.

After each ray was fixed, the position of the tendons which cross the metatarsophalangeal joint was photographed, with the metatarsophalangeal joint both in a neutral position and in positions of dorsal extension, plantarflexion, and superior dislocation. Fluoroscopy was used in order to determine the initial center of rotation of the metatarsophalangeal joint. Subsequently, a Weil osteotomy was performed on half of the rays and a triple Weil osteotomy on the remaining ones.

The Weil osteotomy was performed using a standard thickness saw blade and starting 1-2 mm below the dorsal border of the articular cartilage, intra-articularly. The level of inclination of the osteotomy was about 25^o^ relative to the longitudinal axis of the metatarsal and approximately 10^o^ relative to the weight-bearing surface [15]. When performing the triple Weil osteotomy, a saw blade with a length of 15 mm, a thickness of 0.35 mm, and a width of 10 mm, was used. The first oblique cut was done parallel to the frontal plane and obliquely to the sagittal plane at an angle of 45^o^-60^o^. The second cut was done in the frontal plane and from a dorsal to a plantar direction. Finally, the third oblique cut, was done at the dorsal border of the proximal metatarsal fragment and was absolutely parallel to the first cut [16]. Both osteotomies were fixed with smooth K-wires or Herbert-type cannulated screws. The way of fixation did not affect the parameters of our study, because in the equilibrium system the metatarsal had no contact with the ground, and therefore the latter did not exert any force that could affect the stability of the osteotomy.

After performing each of the two osteotomies, the position of the tendons of the interosseous muscles was controlled again. The final center of rotation of the metatarsophalangeal joint and, therefore, the change of position of the metatarsal head in relation to the diaphyseal longitudinal axis was also determined, through selected coordinated points in views with the use of C-arm.

**Figure 4:**
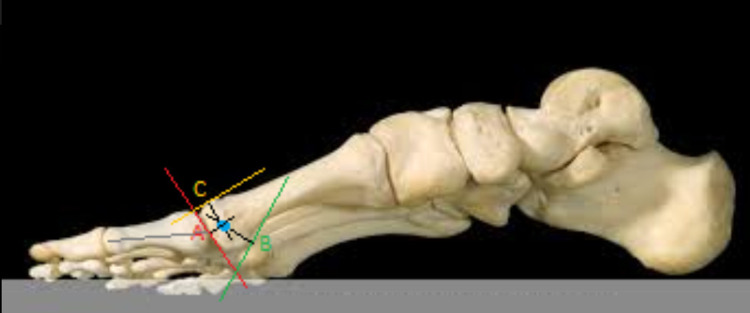
The determination of the center of rotation of the metatarsophalangeal joint was achieved through the use of three points on the metatarsal head. Point A corresponded to the central point of the articular surface. Point B corresponded to the plantar and more proximal edge of the head. Point C corresponded to the most dorsal edge of the head.

The geometric axiom used for the determination of the center of rotation states that, if on the circumference of a circle formed at the sagittal plane, we consider two points and draw their tangents, then the vertical lines in the tangents inwards will always meet at the center of the circle. This axiom also applies to elliptical surfaces, such as the metatarsal head (Figure 4). Based on the above axiom, in the images taken with C-arm pre- and postoperatively, three coordinate reference points were defined. Point A corresponded to the central point of the articular surface of the metatarsal head, which is in the same straight line as the longitudinal axis of the proximal phalanx. Point B corresponded to the lower (plantar) and more proximal edge of the head, before its transition to the plantar surface of the neck. Finally, point C corresponded to the most dorsal edge of the head. In this simplified model, forming the perpendiculars of the tangents of points A, B, and C and extending them inside the head, the point where they meet, gives us each time the center of rotation of the metatarsophalangeal joint (Figure 4).

**Figure 5:**
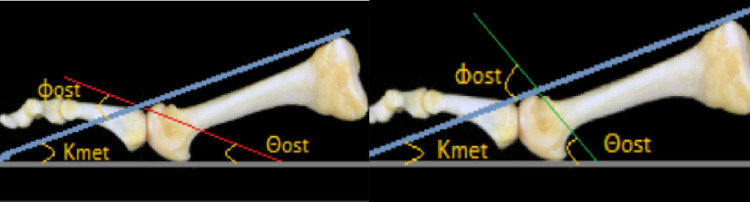
Figure 5. A: Weil osteotomy. B: Triple Weil osteotomy. Red line: Weil osteotomy angle; green line: triple Weil osteotomy angle; blue line: position of the first K-wire, parallel to the dorsal surface of the metatarsus; gray line: position of the second K-wire, parallel to the ground level; Κmet: the inclination of the metatarsal.

To calculate the actual plantar displacement of the metatarsal head relative to the ground, after completion of each osteotomy, two thin 1.6 mm guide K-wires were used. The first one was placed parallel to the dorsal surface of the metatarsal. The second one was placed parallel to the ground. Special care was taken so that the metatarsal and the guide wires were perpendicular to the C-arm beam (we used for this purpose the laser viewfinder of the machine). Lateral views were then taken, which were printed on paper, followed by a geometric analysis of each of the two studied osteotomies. Based on a study by Grimes and Coughlin [17], the level of the osteotomy, the inclination of the metatarsal, and the ground level (plantar reference plane) form a triangle, the sum of the vertices of which is equal to 180 degrees. The missing angle of the triangle and the angle of the osteotomy give a sum of 180 degrees (Figures 5A-5B).

If these equations are combined:

180 = Θost + Κmet + (180 - Φost)

Θost = Φοst - Κmet

Where, Kmet is the inclination of the metatarsal, Φost is the angle of osteotomy indicated with respect to the dorsal surface of the metatarsal, and Θost is the angle of osteotomy indicated with respect to the plantar reference plane.

**Figure 6:**
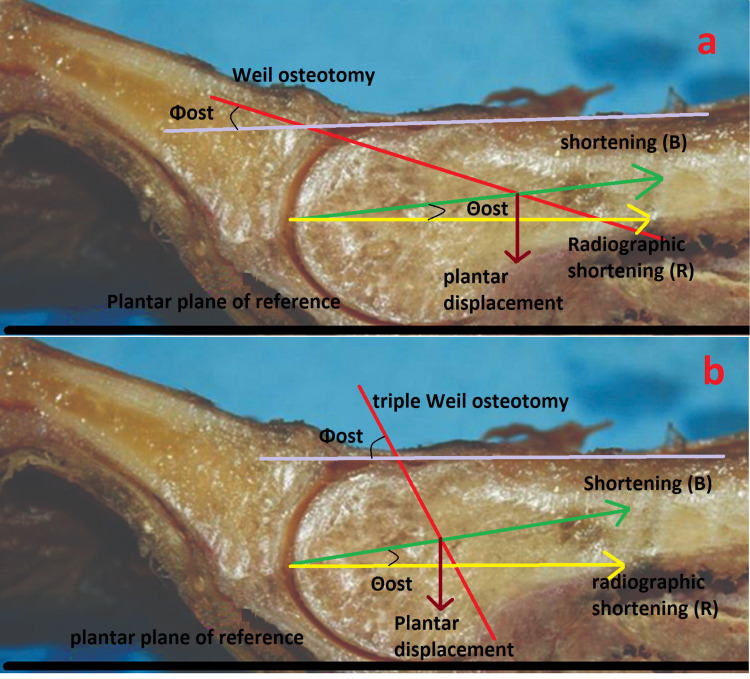
Weil osteotomy (A) and triple Weil osteotomy (B) The first panel shows a Weil osteotomy, where the proximal displacement of the head fragment is calculated using the most distal edge of the proximal fragment as a reference. The second panel shows triple Weil osteotomy, where the proximal displacement of the metatarsal head is measured by the thickness of the bone fragment removed by the second cut. In both cases, the shortening of the metatarsal (B) is along its longitudinal axis. The radiographic shortening (R) is along the plane of reference. Θost: osteotomy angle.

Always using the mathematical equations from the study of Grimes and Coughlin [17], as the head fragment moves along the osteotomy at an angle equal to Θost relative to the ground level, plantar displacement of the head fragment (Π) forms the hypotenuse of a triangle. The shortening of the metatarsal (B) along its longitudinal axis and the radiographic shortening (R) form the other two legs of the triangle (Figures 6A-6B).

The plantar displacement of the head fragment (Π) is given by the equation:

Π = proximal displacement of the head fragment Χ sine(Φost - Κmet)

It is worth noting that the proximal displacement of the head fragment, in the case of the Weil osteotomy, is calculated using the most distal edge of the proximal fragment as a reference (Figure 6A). In the case of the triple Weil osteotomy, the proximal displacement of the metatarsal head is measured by the thickness of the bone fragment removed by the second cut (Figure 6B). This measurement was performed using a digital Vernier caliper.

 Data analysis

Statistical analyses were conducted to investigate the position’s change of the center of rotation of the MTP joint, using SPSS Statistics, version 21 (IBM Corp., Armonk, NY). Student’s t-tests were used to compare the differences between the preoperative and postoperative measurements. The effect of the change of the center of rotation on the orientation of the tendons of the interosseous was analyzed using analysis of variance (ANOVA) with repeated measurements. The deﬁnitions of the different centers of rotation, before and after each osteotomy, were the ﬁxed effects, and rays (2nd, 3rd, 4th) and trials were random effects. The level of significance was defined using a p-value of less than 0.05. Results are presented as means ±95% confidence limits.

## Results

Using the fluoroscopy and taking into account the three reference points on the metatarsal head we were able to determine the center of rotation of the metatarsophalangeal joint. As a result, we noticed that the metatarsophalangeal joint’s center of rotation after the Weil osteotomy is displaced proximally and plantarly by an average of 3.5 ± 1.64 mm compared to the center of rotation in the control group, and by an average of 3.7 ± 1.52 mm compared to the center of rotation in the triple Weil osteotomy group. In both cases, the numerical values ​​indicate a statistically significant difference (p<0.05). Furthermore, comparing the center of rotation’s position of the metatarsophalangeal joint before and after the triple Weil osteotomy, we could not detect a statistically significant difference (p=0.690). Indeed, the center of rotation in the triple Weil osteotomy group was proximally and plantarly displaced, equal to 0.817 ± 0.12 mm on average, relative to the center of rotation in the control group. However, this difference was not statistically significant (Table 1).

**Table 1:** Plantar translation of the metatarsal head (mm) by every osteotomy. The total number of studied specimens: six (three in each group). The total number of studied rays: 18 (six 2nd ones, six 3rd ones, and six 4th ones).

Table 1			
OSTEOTOMY WEIL GROUP (p value)			
	Specimen no1	Specimen no2	Specimen no3
2^nd^ ray	3.93 ± 1.19	3.79 ± 0.61	3.09 ± 1.69
3^rd^ ray	2.23 ± 0.93	3.01 ± 1.83	2.44 ± 1.90
4^th^ ray	3.45 ± 1.52	3.15 ± 1.75	3.53 ± 1.71
TRIPLE WEIL OSTEOTOMY GROUP (p value)			
	Specimen no1	Specimen no2	Specimen no3
2^nd^ ray	1.16 ± 0.32	0.93 ± 0.38	0.82 ± 1.45
3^rd^ ray	0.53 ± 1.14	0.50 ± 1.20	0.46 ± 1.39
4^th ^ray	0.89 ± 0.05	1.12 ± 0.13	0.95 ± 1.02

**Figure 7:**
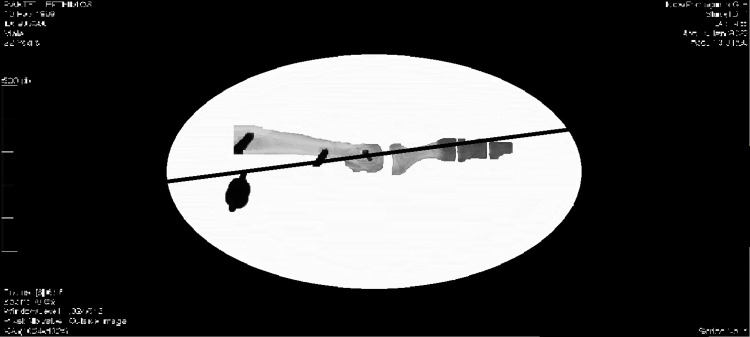
Using a K-wire as a guide for the position of the tendons of the interosseous, after the Weil osteotomy the guide wire was above the metatarsal longitudinal axis.

Using a Kirschner wire as a guide for the position of the tendons of the interosseous, before and after the Weil osteotomy, and comparing the two positions with fluoroscopy, we noticed that after the Weil osteotomy the guide wire was above the metatarsal longitudinal axis. Repeating the same procedure in the case of triple Weil osteotomy, the position’s change of the guide wire does not present a statistically significant difference with respect to the position of the guide wire in the control group (Figure 7 and Table 2).

**Table 2:** Dorsal displacement of the interosseous’s tendons in relation to the metatarsal longitudinal axis (mm) by every osteotomy. The total number of studied specimens: six (three in each group). The total number of studies rays: 18 (six 2nd ones, six 3rd ones, and six 4th ones).

Table 2			
OSTEOTOMY WEIL GROUP (p value)			
	Specimen no1	Specimen no2	Specimen no3
2^nd^ ray	1.33 ± 1.12	1.69± 1.05	2.09 ± 1.06
3^rd^ ray	2.23 ± 1.09	1.37 ± 0.17	1.18 ± 0.21
4^th^ ray	1.44 ± 0.66	1.39 ± 1.29	1.95 ± 1.48
TRIPLE WEIL OSTEOTOMY GROUP (p value)			
	Specimen no1	Specimen no2	Specimen no3
2^nd^ ray	0.05 ± 1.18	0.15 ± 0.53	0.08 ± 1.00
3^rd^ ray	0.03 ± 0.12	0.02 ± 0.27	0.06 ± 1.05
4^th ^ray	0.14 ± 0.31	1.12 ± 1.05	0.05 ± 1.14

## Discussion

The Weil osteotomy is a widely used procedure in the treatment of biomechanical metatarsalgia. Despite the easy technique and effectiveness, this osteotomy has got some drawbacks that can affect the final clinical outcome. Complications include recurrence of symptoms, transfer metatarsalgia in adjacent rays, metatarsophalangeal joint stiffness and a floating toe deformity. The latter is the commonest complication with an incidence of 20-36% in the various studies [18, 19]. Studies until today have shown that after the Weil osteotomy in the lesser metatarsals of the forefoot, there is a change in the position of the tendons of the interosseous muscles, as a result of the proximal translation of the metatarsal head and its associated plantarflexion [19-21]. The position’s change of the tendons of the interosseous muscles in relation to the center of rotation of the metatarsophalangeal joint has an important role in the pathogenesis of the floating toe [21]. This fact becomes especially important when the shortening of the metatarsal is more than 3 mm [22]. Thus, the tendons of the interosseous muscles, which normally act as plantar flexors of the metatarsophalangeal joint, are located in a new position, dorsally of the axis of rotation of these joints, resulting in attenuation of their action or its transformation into extensors for the metatarsophalangeal joint [23].

The results of our study showed that there is a statistically significant difference regarding the plantar displacement of the center of rotation of the MTP joint between the rays which the Weil osteotomy was performed in comparison with the rays where Maceira osteotomy was performed. The plantar displacement of the center of rotation as a consequence of the plantar translation of the metatarsal head results in a change in the position of the tendons of the interosseous muscles. Thus, the tendons from their normal position inferior to the metatarsal longitudinal axis, are located in a new dorsal position superior to the center of rotation. Furthermore, we observed that the triple Weil osteotomy causes minimal or no displacement of the metatarsophalangeal joint’s center of rotation and, therefore, the position of the interosseous muscles remains unchanged.

However, many researchers claim that besides the role of the position of the interosseous’s tendons, another important cause of floating toe is the relaxation of the plantar plate of the metatarsophalangeal joint, which occurs after a distal metatarsal shortening osteotomy [24]. This relaxation may lead to a weakening of the inverted windlass mechanism and, then, the loss of contact of the toe with the ground during the third rocker of gait. A limitation of this study consists of the fact that this parameter has not been studied. For this reason, future studies which take into consideration the role of the plantar plate in the pathogenesis of floating toe are essential. 

Other limitations are the relatively small number of feet, as well as the fact that other factors were not studied which could affect the biomechanical behaviour of the MTP joint, such as a pre-existing subluxation, or a simultaneous procedure at the joint (for example, arthrodesis).

## Conclusions

We concluded that the change of the position of the tendons of the interosseous muscles after Weil osteotomy is a condition able to cause a dorsal contraction of the metatarsophalangeal joint by itself. This is not the case for the triple Weil osteotomy, where the normal position of the interossei and consequently the plantarflexory force that they act at the level of the metatarsophalangeal joint are preserved. So the act of a bigger force is required in order to impinge the metatarsophalangeal joint on the dorsal soft tissues.
